# Supplementary material for: Intra-Articular Injection of Autologous Micro-Fragmented Adipose Tissue for the Treatment of Knee Osteoarthritis: A Prospective Interventional Study
Source: J Pers Med. 2023 Mar 10;13(3):504. doi: 10.3390/jpm13030504 (PMC10059754; doi:10.3390/jpm13030504)
Supplement: Supplementary file 1 [file jpm-13-00504-s001.zip › jpm-2236395-Supplementary Figure S1.pdf]

Supplement-Figure S1 Changes on MRI before and after injection of autologous micro-fragmented adipose tissue.

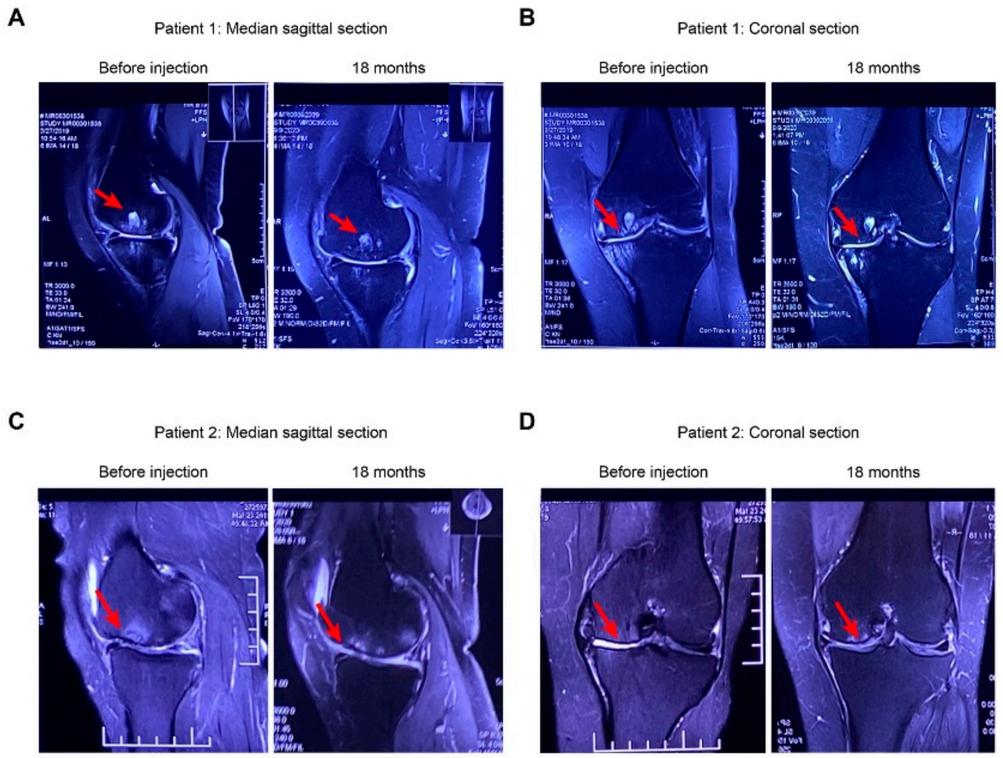

(**A**) & (**B**): Patient 1, female, 53 years old. The images show that the oedema of the femoral condyle and tibial plateau was significantly reduced, the cartilage had thickened, the cartilage signal became continuous, and the Recht grade of the knee cartilage injury had changed from grade II to grade I.

(**C**) & (**D**): Patient 2, female, 60 years old. The images show that the subchondral bone of the femoral condyle was improved by comparing MRI before and 18 months after the injection of autologous micro-fragmented adipose tissue. The cartilage coverage increased and thickened, the knee joint oedema decreased significantly, and the Recht grade of the knee joint cartilage injury changed from grade III to grade II.
